# Single-Stage versus Multi-Stage Intramedullary Nailing for Multiple Synchronous Long Bone Impending and Pathologic Fractures in Metastatic Bone Disease and Multiple Myeloma

**DOI:** 10.3390/cancers15041227

**Published:** 2023-02-15

**Authors:** Aditya V. Maheshwari, Andriy Kobryn, Juhayer S. Alam, Mikhail Tretiakov

**Affiliations:** Department of Orthopaedic Surgery and Rehabilitation Medicine, State University of New York Downstate Health Sciences University, Brooklyn, NY 11203, USA

**Keywords:** intramedullary nailing, metastatic long bone disease, single stage, multiple stage, cardiopulmonary complications

## Abstract

**Simple Summary:**

For patients with advanced metastatic disease presenting with synchronous multiple long bone impending and complete fractures requiring placement of intramedullary nails (IMN), the optimal timing of bone fixation—whether in a single or multiple stages—is still highly debatable. In this work, we compared perioperative outcomes like overall complications, survival, in-hospital death, and postoperative length of stay among others between patients who have undergone single or multi-stage intramedullary nailing procedures for oncological indications. Our findings revealed that single-stage intramedullary nailing synchronous long-bone metastases in select patients does not increase their risk of perioperative complications and in-hospital mortality but leads to earlier postoperative discharge and initiation of rehabilitation. Thus, our results support single-stage multiple nailing as an efficient and viable therapeutic strategy for select patients with multiple long-bone metastases.

**Abstract:**

Purpose: Determine whether perioperative outcomes differ between patients who have undergone single or multi-stage IMN procedures for impending or completed pathologic fractures. Methods: Patients were classified into single-stage single-bone (SSSB), single-stage multiple-bone (SSMB), and multi-stage multiple-bone (MSMB) based on procedure timing and number of bones involved. Outcome variables compared included length of stay (LOS), in-hospital mortality and survival, initiation of rehabilitation and adjuvant therapy, and perioperative complications. Results: There were 272 IMNs placed in 181 patients (100 males, 81 females, 55.2% and 44.8%, respectively) with a mean age of 66.3 ± 12.1 years. MSMB had significantly longer LOS (24.3 ± 14.2 days) and rehabilitation initiation (3.4 ± 2.5 days) compared to SSSB (8.5 ± 7.7 and 1.8 ± 1.6 days) and SSMB (11.5 ± 7.6 and 2.0 ± 1.6 days) subjects, respectively (both; *p* < 0.01). Although total perioperative complication rates in SSMB and MSMB were comparable (33.3% vs. 36.0%), they were significantly higher than SSSB (18%) (*p* = 0.038). MSMB had significantly more (20%) cardiopulmonary complications than SSMB (11.1%) and SSSB (4.5%) (*p* = 0.027). All groups exhibited comparative survivorship (8.1 ± 8.6, 7.1 ± 7.2, and 11.4 ± 11.8 months) and in-hospital mortality (4.5%, 8.9%, and 4.0%) (all; *p* > 0.05). Conclusion: In comparison to MSMB, SSMB intramedullary nailing did not result in higher perioperative complication or in-hospital mortality rates in select patients with synchronous long-bone metastases but led to earlier postoperative discharge and initiation of rehabilitation.

## 1. Introduction

The treatment of long-bone metastases represents a significant challenge to healthcare systems. In the United States, approximately 5 million adults live with bone metastases, resulting in annual medical costs exceeding $12 billion [1,2,3]. Presenting as impending and/or complete pathologic fractures, long-bone metastases are often managed with intramedullary nail (IMN) fixation, which aims to relieve pain, maintain or restore function and mobility, expedite adjuvant treatment, and improve quality of life [4,5,6,7,8,9]. Though commonly performed, IMNs carry significant risks, including fat and tumor emboli, infection, thromboembolism, hardware failure, and cardiopulmonary complications [4,5,6,7,10,11,12,13,14,15,16,17,18,19,20,21].

Multiple synchronous impending and/or complete pathologic long-bone fractures may be present in patients with advanced metastatic disease. In this unique clinical situation, the prospect of undergoing multiple IMN procedures raises concern of increased perioperative adverse events and mortality [7,8,22]. Early reports of increased theoretical thromboembolic events, severe cardiopulmonary complications, and high mortality rates cautioned against simultaneous IMN of multiple bones under one anesthesia [13,23,24,25,26,27,28,29] Therefore, IMN insertion in multiple-stages historically emerged as the preferred therapeutic modality, conducting one bone IMN insertion per surgical setting.

Contrary to IMN insertion in multiple stages, nailing of multiple long bones in a single anesthesia offers numerous promising benefits, including a reduction in the number of trips to the operating room, anesthesia burden, and overall healthcare costs, as well as a mitigation of delays in patient rehabilitation, recovery, discharge, and start of definitive adjuvant treatment [28,29]. Despite contradicting the previously reported high complication and mortality rates encountered with single-stage multiple nailing, recent analyses have been limited by their relatively small sample sizes and lack of adequate control groups [10,28,30,31].

Given the absence of a current consensus on the optimal timing of multiple long-bone nailing, this study aims at comparing outcomes of patients with long-bone metastases undergoing multiple IMN insertions under one or multiple surgical settings. With modern anesthesia, minimally invasive surgical techniques, and improvement in perioperative multidisciplinary medical management, we hypothesized that perioperative outcomes will be comparable between all groups.

## 2. Materials and Methods

### 2.1. Patient Population

**Figure 1:**
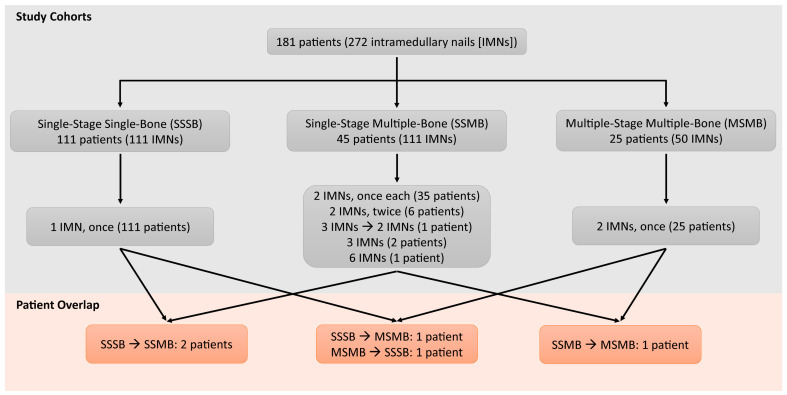
The study cohort and patient group overlap. Seven single-stage single-bone (SSSB) subjects had single intramedullary nails (IMNs) placed twice during separate hospitalizations and were counted as 14 unique events, whereas 13 single-stage multiple-bone (SSMB) patients had their surgery preceded or followed by a single-nail insertion during the same hospitalization and were counted in the SSMB cohort for statistical analysis despite them technically undergoing staged procedures. Four SSMB/MSMB (multiple-stage multiple-bone) patients were also considered in the SSSB group as they had a single nail placed in a different hospitalization either before or after the current hospitalization. There was one patient who had undergone a single- and multi-stage multiple-bone nailing procedures during two separate hospitalizations and was included in both SSMB and MSMB groups.

This study is a retrospective review of a prospectively maintained database, approved by the Institutional Review Board of a single academic urban orthopaedic program. A total of 272 IMNs (181 patients) were inserted between April 2011–December 2020 for metastatic long-bone disease or multiple myeloma (Figure 1). Seventy (38.7%) patients had at least 2 IMNs inserted, accounting for 161 (59.2%) IMNs.

### 2.2. Group Stratification

Based on the number of bones (one vs. multiple) and timing (one stage vs. multi-stage) of IMN insertion during a single hospital admission, patients were classified into 3 groups: (1) Single-stage multiple-bone (SSMB), undergoing IMN insertion of 2 or more bones in one setting/anesthesia, (2) Multiple-stage multiple-bone (MSMB), undergoing staged IMN insertion of multiple bones, one nail at a time, under different anesthesia on separate days, but same admission, and (3) Single-stage single-bone (SSSB), undergoing only one IMN insertion during a single hospital admission (Figure 1). These decisions were made with a multidisciplinary team, taking into consideration the patient’s tumor burden, general medical condition, rehabilitation potential, timing of adjuvant therapeutic modalities, and patient’s and/or their family’s wishes.

### 2.3. Preoperative Optimization

Skeletal survey radiographs were obtained for all patients. The decision for prophylactic IMN was predominantly guided by Mirels’ criteria [32]. Patients were medically optimized and cleared by a multidisciplinary team including, but not limited to, orthopaedic surgery, anesthesia, internal medicine, hematology-oncology, and rehabilitation. If needed, they were pre-transfused to a hemoglobin level of at least 10 g/dL. When appropriate, lactate-levels were obtained between surgeries to ensure adequate resuscitation (below 2 mmol/L). SSMB had their subsequent procedures performed in the same setting only if they were hemodynamically stable and approved by the anesthesia team. Two planned SSMB had their second procedure aborted during the same setting due to hemodynamic issues, but completed the second nailing procedure at a later date and were therefore included in the MSMB group for analysis. Additionally, one patient had planned staged procedures but died before his second procedure and was classified as SSSB.

### 2.4. Primary Demographics

All groups were comparable for age, gender, body mass index (BMI), and primary diagnosis. The most common primary malignancy was multiple myeloma followed by breast and prostate. The most common long bone involved was the femur in all groups, although other differences in procedure location were noted between groups. In contrast to SSSB, most procedures in SSMB and MSMB were performed for impending fractures (Table 1).

The most common combination was femur and humerus in the SSMB and bilateral femur in the MSMB (Table 2). Overlapping patients were placed in more than one group according to their unique clinical course, as well as the timing of their presentation and surgeries, but the data collected were specific for each procedure (or combination of procedures per the defined groups) and admission (Figure 1). Most femur nails (Gamma 3, Stryker Trauma GmbH, Schönkirchen, Germany or TFNA, Depuy-Synthes GmbH, Oberdorf, Switzerland) were 10 mm. Humeral nails (T2 Proximal Humerus Nail (PHN), Stryker Trauma GmbH, Schönkirchen, Germany) were 8 mm in diameter. Diaphyseal reaming was avoided/minimized and only done when the canal was narrow. No canal venting was performed.

### 2.5. Outcome Variables

Extracted variables from electronic medical records included age, gender, BMI, primary malignancy, surgery details, type (impending or pathologic) and location, number of nails placed, perioperative complications, estimated blood loss (EBL), and packed red blood cell (PRBC) units given intraoperatively or immediately after the procedure in the recovery room. Chart review and discharge summaries were queried to extract postoperative complications, date of return to definitive adjuvant therapy (chemotherapy, radiation), date of initiation of rehabilitation, and date of discharge/ death. Length of stay (LOS) was defined as the number of days between the first surgery and discharge, and mortality was labeled as “in-hospital” when death occurred during the same admission. After discharge, if a definitive date of death was not found after querying all available resources, patients were deemed lost to follow-up. Last follow-up was defined as the date of the last documented visit to the clinic or hospital.

### 2.6. Statistical Analysis

Descriptive analyses were performed to summarize patient demographics, operative-variables, and hospital outcomes. Owing to the variability in EBL and PRBC values, extreme outliers were identified and excluded by subtracting or adding 3 times the interquartile range to the 25th or 75th percentiles, respectively. A one-way analysis of variance with post-hoc Tukey was conducted to compare the means of continuous variables among the groups, including patient age, BMI, EBL, PRBC, LOS, return to adjuvant therapy, and initiation of rehabilitation. A Fisher’s exact test with Freeman-Halton extension was performed to compare categorical variables between the study cohorts, including gender, fracture type and location, complications, and death. Survivorship was assessed using a log-rank test with Kaplan–Meier estimates. All statistical analyses were conducted in an SPSS version 28.0 (IBM Corporation, Armonk, NY, USA), using a *p*-value of 0.05 as threshold for statistical significance.

## 3. Results

### 3.1. Perioperative Outcomes

Perioperative outcomes are summarized in Table 3. SSMB and MSMB had comparable total EBL (419 ± 197, and 467 ± 238 Ml) and PRBCs given (1.2 ± 1.3 and 0.8 ± 1.3 units), but differed from the SSSB, which had less EBL (219 ± 134 Ml) and PRBCs given (0.4 ± 0.9) (all; *p* < 0.001). MSMB stayed longer in the hospital (24.3 ± 14.2 days) and were slower to start rehabilitation (3.4 ± 2.5 days) than the SSSB (8.5 ± 7.7 and 1.8 ± 1.6 days) and SSMB (11.7 ± 7.6 and 2.0 ± 1.6 days), respectively (both; *p* < 0.01). No differences were found in postoperative return to adjuvant therapy (SSSB: 25.5 ± 20.3; SSMB: 26.6 ± 23.1; MSMB: 27.4 ± 12.1 days, *p* > 0.05), although several patients had their definitive oncologic care at different institutions which may have dictated the timing.

### 3.2. Postoperative Complications

Adverse outcomes experienced by all patients are detailed in Table 4. Overall complication rates were comparable in the SSMB (15; 33.3%) and MSMB (9; 36.0%) but significantly lower in the SSSB (20; 18.0%) (*p* = 0.038). A total of 10 of 181 (5.5%) patients developed surgical complications (8 SSSB, 2 SSMB, 7.2% and 4.4%, respectively, and none in MSMB), with no significant differences (*p* = 0.408). Thirty-five (19.3%) total medical complications were noted, more so in the SSMB (14 out of 45; 31.1%) and MSMB (9 of 25; 36.0%), than the SSSB (12 of 111; 10.8%) (Table 3; *p* < 0.001). Moreover, the MSMB had a significantly more cardiopulmonary complications (*p* = 0.027) compared to SSMB and SSSB (5 out of 25, 5 out of 45, and 5 out of 111; 20%, 11%, and 4.5% respectively).

### 3.3. Survivorship and In-Hospital Mortality

**Figure 2:**
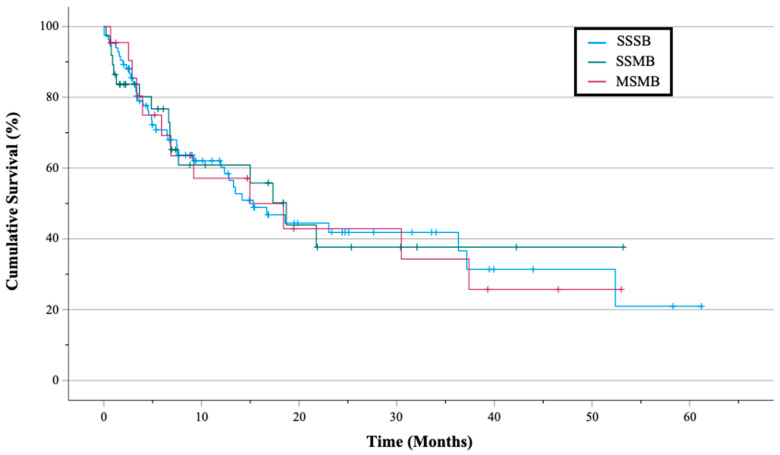
Kaplan–Meier survival curve comparing postoperative survival days of SSSB, SSMB, and MSMB groups (*p* = 0.996).

The median Kaplan–Meier survivorship of SSSB, SSMB, and MSMB was 15.3, 18.7, and 15.0 months, respectively (Figure 2; *p* = 0.996). Overall, 10 (5.5%) in-hospital deaths were documented: 5 of 111 (4.5%) occurred in the SSSB, 4 of 45 (8.9%) in the SSMB, and 1 of 25 (4.0%) in the MSMB (Table 3 and Table 4; *p* = 0.524). No intraoperative death was noted, but one SSSB patient (breast adenocarcinoma), prophylactically managed with unreamed femoral IMN insertion, developed an ST-elevation myocardial infarction during a proximal lag screw placement (Table 4). As per the patient’s and family’s wishes, she underwent neither resuscitation nor prolonged intubation and died 8 h later in the recovery room.

## 4. Discussion

Modern advances in cancer diagnosis and treatment have increased survival rates and resulted in a higher incidence and prevalence of metastatic disease [33,34,35]. In patients with long-bone metastasis, IMN is regarded as the surgical standard of care for impending and/or complete pathologic fracture fixation [36,37,38,39]. Yet, for subjects presenting with multiple synchronous metastatic long-bone fractures, no consensus currently exists as to whether these procedures should be staged or performed under a single surgical anesthesia.

Contrary to early reports documenting high complication and mortality rates, recent preliminary evidence regarding single-stage nailing of multiple long bones has been encouraging [10,23,24,25,26,27,28,29,30,31]. However, these limited small series studies lack several clinically relevant variables, and no study has ever compared SSMB vs. MSMB directly. In 16 SSMB (33 IMNs), of whom 12 (75%) underwent combined femur-humerus nailing, Moon et al. noted in-hospital and intraoperative mortality rates of 18.8% and 6.3%, respectively [28]. In another multivariate survivorship analysis of 927 subjects where 35 (3.8%) underwent SSMB surgery (12 bilateral femora and 16 femur-humerus combinations; 34.4% and 45.7%, respectively), SSMB did not show any decreased survival in this group, although only 60% had IMN fixation [22]. The SSMB cohort in our study showed an 8.9% in-hospital death rate with no difference between MSMB and SSSB, possibly highlighting a significant improvement in survival, mortality and complication rates with the implementation of contemporary surgical techniques, medical therapies, and appropriate patient selection over the years.

Long-bone IMN insertion has been associated with both medical and surgical complications, partially attributed to the patients’ advanced disease and high tumor burden, associated comorbidities, and reduced baseline reserves [4,5,6,7,10,11,12,13,14,15,16,17,18,19,20,21]. Owing to tumor, fat, and thrombotic emboli, cardio-pulmonary complications are often the most concerning and devastating complications in this patient population [13,23,24,25,26,27,28,29,30,31]. Moon et al. noted cardio-pulmonary complications in 9 (56.3%) of 16 patients undergoing multiple IMN insertions in a single surgical-setting, three of whom died prior to discharge [28]. Our overall complication (both medical and surgical) rates were almost double in both multi-nail groups (MSMB: 36.0%; SSMB: 33.3%) compared to the SSSB group (18.0%). However, these differences were mostly in medical complications as surgical complications were similar between all groups. This could be explained by the fact the patients requiring multiple nails (SSMB and MSMB) were relatively sicker with advanced disease. Thus, utmost attention needs to be paid to optimize them for surgeries. Cardio-pulmonary events accounted for a majority (15/35, 43%) of medical complications in our study. Five (11.1%) SSMB had cardio-pulmonary adverse events, none of whom died during their hospital stay. Comparatively, 5 (4.5%) SSSB and 5 (20.0%) MSMB had cardio-pulmonary complications, two of whom (one in each group) died in-hospital (*p* = 0.027). The highest rate of cardio-pulmonary events in the MSMB group could be due to the selection bias as MSMB patients generally had the greatest disease burden of all study groups and thus could only tolerate staged procedures. Also, the MSMB group consisted mostly of bilateral femur nailing (21/25, 84%), the combination with more potential for cardio-pulmonary complications [23,24,25,26,27].

Data on single-stage bilateral femur nailing in metastatic setting is very limited with a high in-patient mortality rate [10,28,29]. Our limited data on 7 SSMB bilateral femora is encouraging as we found comparable rates of overall complications (1 out of 7, or 14.3% vs. 8 out of 21, or 38.1%; *p* = 0.37), and inpatient mortality (none vs. 1, or 4.8%) when compared to MSMB bilateral femora (n = 21) during the same admission (Table 4). There were no surgical complications in each group, and cardiopulmonary complications were also comparable (1 out of 7, or 14.3% vs. 5 out 21, or 23.8%, *p* = 0.52). However, there may be a selection bias and possibility of a type II error, and additional higher-level studies with larger sample size studies are warranted to address the outcomes of single stage bilateral femur nailing.

The SSMB had a shorter LOS as compared to MSMB (11.5 vs. 24.3 days), but comparative to SSSB (8.7 days). Ristevski et al. reported a mean LOS of 26.6 days in bilateral femur cases during the same admission compared to 22.3 days in unilateral cases, but it was not clear whether the procedures in the study were single or multi-staged [10]. Even in traumatic bilateral femur fractures, longer LOS was found in patients undergoing a multi-staged vs. single-stage IMN insertion (28.5 vs. 16.4 days) [40]. Our SSMB were able to initiate postoperative physical therapy earlier (2.03 days) than the MSMB (3.42 days), likely because many MSMB did not start their physical therapy until after their second operation, especially when both lower limbs were involved. Though SSMB were expected to resume definitive adjuvant chemotherapy and/or radiation earlier than the MSMB, we did not find any significant differences between the groups. This may have occurred because many MSMB received adjuvant therapy (specially started radiation to the first operative site) between surgeries. Moreover, several of our patients had their primary medical and oncological care at a different institute and were referred to us only for surgical intervention. Thus, their adjuvant treatment was dependent on several extrinsic factors which impacted the time to resumption of adjuvant therapy.

Our study has several limitations, which may prevent the generalization of our findings. Despite being a retrospective review of a prospectively maintained database, we had a relatively small sample size and some variables were missing. Specifically, definitive death dates, resumption of adjuvant therapy, and initiation of rehabilitation were only available on 40%, 60%, and 80% of patients, respectively. Although MSMB surgery should have the highest cost due to longer LOS as well as multiple trips to the operating room, no actual cost-analysis was performed in this study, as the total cost of cancer treatment depends on multiple factors, of which surgery-related costs are only a small fraction. Due to numerous possible permutations and combinations in the patient population and their presentation with multiple bone involvement in different time frames, some overlap invariably existed between SSSB, SSMB, and MSMB, complicating group stratification, data collection, and statistical analyses. Moreover, in the absence of current guidelines and protocols, the final decision regarding the staging may have contributed to a systematic selection bias, as not all patients were deemed candidates for single-stage fixation. Finally, most study participants were diagnosed with multiple myeloma. This malignancy is prevalent in our community and has a similar surgical treatment compared to other long-bone metastatic diseases; however, its prognosis may be different from the latter. While randomization would have ultimately added more strength to this study, it was not possible due to ethical concerns. Despite such limitations, our findings add value to the growing literature supporting the use of multiple nails in one surgical setting. To the best of our knowledge, ours is the first study of its kind to compare SSSB, SSMB, and MSMB groups with multiple outcome variables.

## 5. Conclusions

SSMB IMN insertion in impending/pathological metastatic fracture did not exhibit inferior survivorship, complications, and in-hospital mortality rates compared to MSMB subjects. Most complications were medical (majority cardio-pulmonary) and thus warrant the need for meticulous optimization of these patients before surgery. SSMB IMN insertion also reduced the LOS in hospital and the time to initiation of rehabilitation. Our findings support single-stage multiple nailing as an efficient and viable therapeutic strategy in select patients. Though ours is the first study of its kind with encouraging results, further investigations are warranted to determine an optimal protocol for the surgical management of these complex and medically fragile patients.

## Figures and Tables

**Table 1:** Demographic characteristic of study population.

	Total Patients (N = 181) IMN (N = 272)	SSSB Patients (N = 111) IMNs (N = 111)	SSMB Patients (N = 45) IMNs (N = 111)	MSMB Patients (N = 25) IMNs (N = 50)	*p*-Value
Age (years) ^‡^	66.3 ± 12.1 [32–96]	66.9 ± 12.4 [38–93]	64.9 ± 12.3 [32–93]	66.4 ± 10.6 [42–96]	0.661
Sex ^†^					0.320
Male	100 (55.2%)	66 (59.5%)	21 (46.7%)	13 (52.0%)	
Female	81 (44.8%)	45 (40.5%)	24 (53.3%)	12 (48.0%)	
BMI (kg/m^2^) ^‡^	27.1 ± 6.3	27.8 ± 7.1	26.4 ± 5.4	25.4 ± 4.31	0.160
Fracture Type ^†^					<0.001
Impending	172 (63.2%)	55 (49.6%) ^b,c^	76 (68.5%) ^a^	41 (82.0%) ^a^	
Pathologic	100 (36.8%)	56 (50.4%) ^b,c^	35 (31.5%) ^a^	9 (18.0%) ^a^	
Metastasis Location ^†^					<0.001
Femur	177 (65.1%)	76 (68.7%) ^b,c^	55 (49.6%) ^a,c^	46 (92.0%) ^a,b^	
Humerus	79 (29.0%)	32 (28.8%) ^c^	44 (39.6%) ^c^	3 (6.0%) ^a,b^	
Tibia	6 (2.2%)	1 (0.9%)	4 (3.6%)	1 (2.0%)	
Fibula	2 (0.7%)	2 (1.8%)	0 (0.0%)	0 (0.0%)	
Radius	6 (2.2%)	0 (0.0%) _b_	6 (5.4%) ^a^	0 (0.0%)	
Ulna	2 (0.7%)	0 (0.0%)	2 (1.8%)	0 (0.0%)	
Primary Diagnosis ^†^					0.497
Multiple Myeloma	84 (46.4%)	47 (42.3%)	24 (53.3%)	13 (52.0%)	
Breast	26 (14.3%)	13 (11.7%)	10 (22.2%)	3 (12.0%)	
Prostate	22 (12.2%)	14 (12.6%)	4 (8.9%)	4 (16.0%)	
Lung	13 (7.2%)	9 (8.1%)	1 (2.2%)	3 (12.0%)	
Renal	10 (5.5%)	8 (7.2%)	2 (4.4%)	0 (0.0%)	
Colon	4 (2.2%)	4 (3.6%)	0 (0.0%)	0 (0.0%)	
Lymphoma	3 (1.7%)	3 (2.7%)	0 (0.0%)	0 (0.0%)	
Hepatocellular	3 (1.7%)	1 (0.9%)	2 (4.4%)	0 (0.0%)	
Other	16 (8.8%)	12 (10.8%)	2 (4.4%)	2 (8.0%)	

BMI: Body Mass Index; IMN: Intramedullary Nail; MSMB: Multiple-Stage Multiple-Bone; SD: Standard Deviation; SSMB: Single-Stage Multiple-Bone; SSSB: Single-Stage Single-Bone. ^‡^ Reported as mean and standard deviation: Mean ± SD [range]. ^†^ Reported as sample size and percentage: N (%). Each column is assigned a letter alphabetically. The presence of a superscript letter indicates a significant difference between the column in which the superscript letter appears and the column correlated to the superscript letter. The column shaded in gray represents combined values of the SSSB, SSMB, and MSMB groups and was not used for statistical comparison amongst the groups.

**Table 2:** Distribution of bones in patients who underwent multiple intramedullary nail insertion.

IMN Combination	SSMB Patients (N = 45) *	MSMB Patients (N = 25)
Femur and Humerus	32	3
Bilateral Femur	7	21
Bilateral Humerus	3	0
Femur and Tibia	3	1
Femur and Radius	2	0
Femur, Humerus, and Tibia	1	0
Femur, Humerus, and Radius	1	0
Humerus and Radius	1	0
Bilateral Humerus and Femur	1	0
Femur, Humerus, Bilateral Radius, and Bilateral Ulna	1	0
Total surgical/anesthesia settings	52 *	50
Total nails placed	111 *	50

IMN: Intramedullary Nail; MSMB: multiple-stage multiple-bone group; SSMB: single-stage multiple-bone group. * Seven patients had multiple IMNs placed in multiple bones twice, accounting for 52 surgical settings in total.

**Table 3:** Perioperative data of patients with metastatic long-bone disease who underwent intramedullary nail fixation in different study groups.

	SSSB Patients (N = 111)	SSMB Patients (N = 45)	MSMB Patients (N = 25)	*p*-Value
EBL (mL) ^‡^	219 ± 134 ^b,c^	419 ± 197 ^a^	467 ± 238 ^a^	<0.001
PRBC (units) ^‡^	0.4 ± 0.9 ^b^	1.2 ± 1.3 ^a^	0.8 ± 1.3	<0.001
Length of Stay (days) ^‡^	8.5 ± 7.7 ^c^	11.7 ± 7.6 ^c^	24.3 ± 14.2 ^a,b^	<0.001
Survival (months) ^‡^	8.1 ± 8.6	7.1 ± 7.2	11.4 ± 11.8	0.424
Adjuvant Therapy (days) ^‡^	25.5 ± 20.3	26.6 ± 23.1	27.4 ± 12.1	0.933
Initiation of Rehabilitation (days) ^‡^	1.8 ± 1.6 ^c^	2.0 ± 1.6 ^c^	3.4 ± 2.5 ^a,b^	0.002
Medical Complications ^†^	12 (10.8%) ^b,c^	14 (31.1%) ^a^	9 (36.0%) ^a^	<0.001
Surgical Complications ^†^	8 (7.2%)	2 (4.4%)	0 (0.0%)	0.408
Total Complications ^†,^*	20 (18.0%) ^b,c^	15 (33.3%) ^a^	9 (36.0%) ^a^	0.038
Death ^†^	5 (4.5%)	4 (8.9%)	1 (4.0%)	0.524

EBL: Estimated Blood Loss; MSMB: Multiple-Stage Multiple-Bone; PRBC: Packed Red Blood Cells; SSMB: Single-Stage Multiple-Bone; SSSB: Single-Stage Single-Bone. ^‡^ Reported as mean and standard deviation: Mean ± SD. ^†^ Reported as sample size and percentage: N (%). * Numbers do not necessarily add up since one SSMB patient had both a medical and a surgical complication. Each column is assigned a letter alphabetically. The presence of a superscript letter indicates a significant difference between the column in which the superscript letter appears and the column correlated to the superscript letter.

**Table 4:** Medical and surgical complications.

Sex	Age	Group	Location	Primary Tumor	Complication	In-Hospital Mortality
**Medical Complications ^a^**						
Female	46	SSSB	Femur	Breast	Deep vein thrombosis and pulmonary embolism	
Male	68	SSSB	Femur	Multiple myeloma	Atrial fibrillation	
Female	55	SSSB	Humerus	Breast	Septic shock from urinary tract infection	44 days postoperatively
Male	85	SSSB	Femur	Prostate	Urinary tract infection	
Male	80	SSSB	Femur	Colon	Urinary tract infection	
Male	49	SSSB	Femur	Pleomorphic sarcoma	Progression of existing deep vein thrombosis	
Female	68	SSSB	Femur	Multiple myeloma	Urinary tract infection	
Male	67	SSSB	Femur	Lung	Pulmonary embolism	
Female	51	SSSB	Femur	Breast	Intraoperative ST-elevation myocardial infarction	8 h postoperatively *
Female	57	SSSB	Femur	Leiomyosarcoma	Pulmonary embolism	1 day postoperatively
Male	83	SSSB	Femur	Renal	Intra-pelvic bleed requiring embolization	17 days postoperatively due to respiratory failure
Male	60	SSSB	Femur	Prostate	Sepsis from urinary tract infection, cerebrovascular accident (CVA)	79 days postoperatively due to CVA
Male	71	SSMB	Femur; Humerus	Multiple myeloma	Pneumonia requiring intubation	
Male	81	SSMB	Femur; BL Humerus	Prostate	Disseminated intravascular coagulation	
Female	66	SSMB	Femur; Humerus; BL Ulna; BL Radius	Multiple myeloma	Clostridium difficile infection	
Female	60	SSMB	Femur; Humerus	Multiple myeloma	Cervical spinal instability leading to cord compression with quadriparesis, requiring urgent neurosurgical decompression and fixation	
Female	38	SSMB	Femur; Humerus	Breast	Pulmonary embolism	
Female	71	SSMB	Femur; Humerus	Multiple myeloma	Pneumonia	
Male	65	SSMB	Femur; Humerus	Multiple myeloma	Disseminated intravascular coagulation	7 days postoperatively
Male	72	SSMB	Femur; Radius	Multiple myeloma	Urinary tract infection	
Male	68	SSMB	Femur; Humerus	Liver	Sepsis from urinary tract infection, gastrointestinal (GI) bleed	30 days postoperatively due to GI bleed
Male	83	SSMB	Femur; Humerus	Multiple myeloma	Multiorgan failure from progression of disease	20 days postoperatively
Female	52	SSMB	Humerus; radius	Multiple myeloma	Multiorgan failure from progression of disease	23 days postoperatively
Female	64	SSMB	BL Femur	Breast	Hypotension	
Female	64	SSMB	Femur; Tibia	Breast	Urinary tract infection	
Female ^†^	57	SSMB	Femur; Humerus	Breast	Hypotension and bradycardia	
Female	74	MSMB	BL Femur	Breast	Bilateral pleural effusion	
Female	52	MSMB	Femur; Tibia	Thyroid	Thyroid storm	
Female	78	MSMB	BL Femur	Multiple myeloma	Hypotension	
Female	74	MSMB	BL Femur	Multiple myeloma	Respiratory distress	
Female	68	MSMB	BL Femur	Lung	Pulmonary embolism, multiorgan failure due progression of disease	22 days postoperatively due to progression of disease
Male	57	MSMB	BL Femur	Multiple myeloma	Pneumonia	
Male	70	MSMB	BL Femur	Prostate	Urinary tract obstruction and infection	
Female	56	MSMB	BL Femur	Multiple myeloma	Acute kidney injury	
Male	60	MSMB	BL Femur	Lung	Urinary tract infection	
**Surgical Complications ^b^**						
Female	77	SSSB	Humerus	Breast	Proximal locking screw back-out, not needing further surgery (asymptomatic)	
Male	70	SSSB	Femur	Multiple myeloma	Progression of bisphosphonate induced subtrochanteric fracture; treated conservatively with vitamin D and calcium healed uneventfully at one year	
Female	64	SSSB	Humerus	Multiple myeloma	Intraoperative distal humerus periprosthetic fracture; requiring additional plating	
Male	62	SSSB	Humerus	Renal	Cement extrusion in the joint and the fracture site acting as large loose body requiring immediate arthrotomy and removal	
Male	57	SSSB	Humerus	Multiple myeloma	Painful locking screw back-out, requiring removal at 2 years	
Male	70	SSSB	Femur	Colon	Failed hardware and fracture non-union due to progression of disease requiring revision nailing with a distal plate combo and cementation at 2 years	
Male	57	SSSB	Humerus	Multiple myeloma	Nondisplaced distal humerus periprosthetic fracture after 10 weeks; treated conservatively with radiation and splint	
Male	74	SSSB	Femur	Multiple myeloma	Failed hardware with progression of fracture and disease; refused further treatment	
Female	44	SSMB	Femur; Humerus	Breast	Transient radial nerve palsy treated conservatively, recovered	
Female ^†^	57	SSMB	Femur; Humerus	Breast	Cement extrusion in the adjacent joint requiring immediate arthrotomy and removal	

MSMB: Multiple-Stage Multiple-Bone; SSMB: Single-Stage Multiple-Bone; SSSB: Single-Stage Single-Bone. BL: bilateral. ^†^ Same patient with medical and surgical complications. * This patient was ‘do not resuscitate/do not intubate’ and the family did not want any resuscitation measure. ^a^ Hypotension noted as complication only if medical intervention was needed. ^b^ Some cement extrusion was common in fracture site for humeral nails. It was not considered a surgical complication unless it needed an unplanned intervention.

## Data Availability

The data presented in this study are available on request from the corresponding author. The data are not publicly available due to privacy and ethical considerations.
